# Depression and Suicide Risks Among Nurses at Sahloul University Hospital in Sousse (Tunisia)

**DOI:** 10.1192/j.eurpsy.2025.1309

**Published:** 2025-08-26

**Authors:** J. Ben Khelifa, F. Chelly, N. Bel Hadj Chabbah, A. Fki, C. Sridi, N. Ben Gadha, S. Ksibi

**Affiliations:** 1 Department of Occupational Medicine, Sahloul University Hospital; 2 Faculty of Medicine Ibn Al Jazzar, University of Sousse; 3 Research laboratory LR 19SP03, Sousse, Tunisia

## Abstract

**Introduction:**

Depression and suicide risk are particularly concerning mental health issues in the workplace, especially among nurses. This group of workers often faces high levels of stress and pressure due to the demanding nature of their job.

**Objectives:**

Our study focuses specifically on describing depression and suicide risk among nurses.

**Methods:**

This is a descriptive study, conducted during January and February 2023, involving 55 nurses working at Sahloul University Hospital in Sousse. Data were collected through a self-administered questionnaire and the Beck Depression Inventory which assesses subjective aspects of depression.

**Results:**

51% of the participants report that working as a nurse constitutes a significant mental burden. During their hospital careers, 47% have faced violence from patients’ family members or companions. (43%) experienced violence from colleagues, mainly in the form of verbal aggression. Nearly all the nurses surveyed (94%) reported demotivation toward their work, with (97.9%) attributing it to work overload and (39.4%) to workplace conflicts. According to the Beck scale, (38%) of the nurses did not present depression, moderate depression was found in (58%) of participants and only 4% had severe depression. Reduced job performance was noted in (16%) of cases. Concerning suicidal ideation, it was reported by only 2 nurses.

**Conclusions:**

Early identification of mental distress and the implementation of appropriate psychological support strategies, are crucial to mitigating these risks. Recognizing psychological risks in hospitals not only improves the well-being of nurses but also enhances the quality of care they provide to patients.

**Disclosure of Interest:**

None Declared

